# A population-based study on the treatment and outcome of enterococcal prosthetic joint infections. A consecutive series of 55 cases

**DOI:** 10.7150/jbji.35683

**Published:** 2019-11-07

**Authors:** Olof Thompson, Magnus Rasmussen, Anna Stefánsdóttir, Bertil Christensson, Per Åkesson

**Affiliations:** 1 Lund University, Department of Clinical Sciences Lund, Division of Infection Medicine and Skåne University Hospital, Department of Infectious Diseases, Lund, Sweden;; 2 Lund University, Department of Clinical Sciences Lund, Division of Orthopedics and Skåne University Hospital, Department of Orthopedics, Lund, Sweden.

**Keywords:** prosthetic joint infection, *Enterococcus faecalis*, * Enterococcus faecium*, debridement, outcome, treatment

## Abstract

AIM: Enterococci cause 2-11% of all prosthetic joint infections (PJI) and are generally considered difficult to treat. However, study-results are not consistent. In this study we present a population-based case series of 55 cases with enterococcal PJI, investigating treatment and outcome, as well as describing the affected patient population regarding demography and co-morbidities.

METHODS: We identified all enterococcal PJIs in the Region of Skåne, Sweden, during a five-year period (2011-2015) and reviewed the patients' medical records.

RESULTS: Fifty-five enterococcal PJIs were found. Enterococcus faecalis was the most frequently isolated species (84%), and poly-microbial infections were common (64%). Treatment with intention to cure was given to 43 (78%) cases. Debridement with retention of the implant and antibiotics (DAIR) was the most common surgical treatment strategy (71%), with a cure-rate of 72%. Overall cure-rate was 67%, and in cases where cure was intended, this was achieved in 80%.

CONCLUSIONS: When cure is aimed for, the prognosis for enterococcal PJI is not so poor, and DAIR treatment can provide adequate results in many cases.

## Introduction

Prosthetic joint infections (PJIs) due to enterococci account for 2-11% 1-4 of all PJIs and are regarded as difficult to treat 5. Enterococci are biofilm forming when attached to the surface of foreign body materials and have a high degree of antimicrobial resistance 6. These features and the fact that the affected patient population is generally elderly with complicating co-morbidities have been suggested to contribute to the difficulties in treatment 7,8.

Enterococcal PJIs are frequently poly-microbial, and have been associated with a higher failure rate than both staphylococcal and streptococcal PJIs 7,9,10. Two-stage exchange of infected prostheses is therefore the generally recommended surgical treatment modality, and one-stage exchange is considered contraindicated 5,11. However, evidence to support these recommendations is weak. A few published reports regarding enterococcal PJIs 7,12-14 present varying results, lending partial support to the notion of “difficult-to-treat”, but randomized trials are lacking. As for the use of surgical debridement with implant retention, which is the preferred modality for most staphylococcal and Gram-negative early or acute hematogenous PJIs, no clear recommendation exists as of yet. Reports on this procedure in enterococcal PJI, however, have presented acceptable results in selected patient populations 15,16.

In this study we present a population-based case series of 55 cases with enterococcal PJI. Our main goal was to investigate the treatment alternatives and outcome, as well as to describe the affected patient population with regard to demography and co-morbidities.

## Materials and method

### Design and data collection

Adult patients with enterococcal PJI diagnosis between January 1:st 2011 and December 31:st 2015 were identified through the clinical microbiology laboratory, Skåne, Sweden. Culture results from joint aspirate or tissue-samples with growth of enterococci were obtained, after which patient medical records were retrospectively reviewed. The Department of clinical microbiology, Skåne, is the only laboratory for clinical microbiology samples in the county, and services all healthcare units. Thus, all patients with a diagnosis of enterococcal PJI during the study-period were likely detected in this consecutive case series. The regional ethics review-board approved the study on June 14 2016 (entry no 2016/343).

### Definitions

Diagnosis of PJI required clinical findings in a prosthetic joint concurrent with infection (eg. joint pain, swelling, wound discharge, sinus tract, pus surrounding the prosthesis, fever or other signs of inflammation) and the presence of joint fluid or tissue cultures growing enterococci. Isolation of enterococci in two or more joint fluid or tissue samples was regarded as significant, as was monomicrobial growth from a single sample. Patients treated partly outside of Skåne were excluded to avoid selection bias as Skåne University Hospital is the regional referral center for complicated PJI.

Episodes were defined as beginning at the isolation of the pathogen and ending at the termination of the antimicrobial treatment. All surgical treatments within this time-frame were regarded as part of the episode. Episodes were classified as early if onset of inflammatory symptoms occurred less than 30 days after insertion of the prosthesis and as delayed if more than 30 days had passed from insertion to the presentation of symptoms. Cases with a sudden onset of inflammatory symptoms well beyond the postoperative period with a previously well-functioning joint were classified as acute hematogenous.

Treatment outcome was deemed as successful if, at one year after the end of the episode, a prosthetic joint was still in place without inflammatory signs or symptoms. Episodes with chronic antimicrobial suppression therapy, permanent removal of the implant, amputation, relapse or death from infection were deemed as failures. Relapse was defined as reoccurrence of the same enterococcal species in the joint after termination of antibiotic treatment. Re-infection with new pathogens was not considered failure, and neither was repeated surgical debridement to control the infection.

### Statistical analysis

Continuous variables are presented as means and standard deviations (SDs) or as medians and interquartile ranges (IQRs). Students' *t*-test and Mann-Whitney U-test were used for normally and non-normally distributed variables respectively. Categorical variables are presented as counts and percentages. Analyses were performed using Fisher´s exact test. The cumulative probability of cure was estimated using the Kaplan-Meier survival method (log-rank test). Significance was set at *p* ≤ 0.05. Statistical analysis was performed using the statistical software IBM SPSS v25.

## Results

### Frequencies and demographics

**Figure 1:**
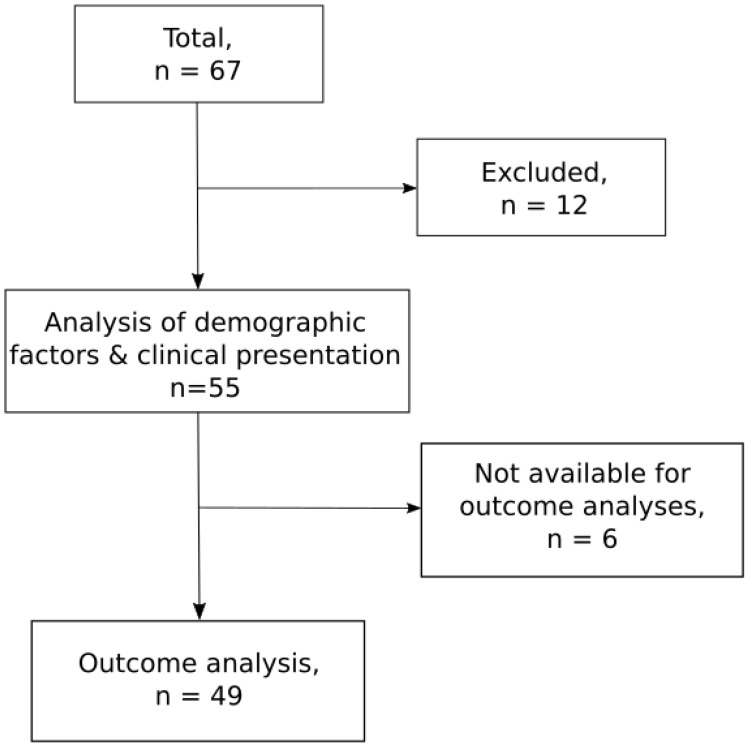
** Flow chart of enterococcal PJI.** Twelve cases failed to meet the inclusion criteria due to non-significant growth (n=6), partial treatment outside Skåne (n=2), episode start before study-period (n=2) and not being prosthesis-related (n=2). Six cases were excluded from the outcome analyses due to death within a year from other causes (n=2) and follow up < one year (n=4).

67 PJI episodes in 65 patients were reviewed for inclusion. 12 episodes were excluded from further analysis due to non-significant growth (n=6), treatment outside of Skåne (n=2), not prosthesis-related (n=2) and episode start before study period (n=2). 55 PJI episodes in 54 patients fulfilled the inclusion criteria (**Figure 1**). During the study-period Skåne had a mean population of approximately 1 275 000 inhabitants, giving an approximate incidence of enterococcal PJI of 0.86/100 000 per year. Episodes were evenly distributed over the five-year study period (9-13 episodes per year). At the time of diagnosis, the median age was 77 (IQR: 74-87) years, and 44% were female. 28 (51%) patients had one or more co-morbidities (table 1).

### Microbiology and presentation

*Enterococcus faecalis* was isolated in 46 cases and *Enterococcus faecium* in 9 cases, with both species isolated in one case. None of the *E. faecium* isolates were vancomycin-resistant (VRE). One episode due to *Enterococcus casseliflavus* was encountered. 35 (64%) episodes were polymicrobial. Co-infecting pathogens were predominantly staphylococci (*Staphylococcus aureus*, n=14, coagulase-negative staphylococci, n=17) and various Gram-negative rods (n=8). In 15 episodes more than one co-infecting pathogen was found. No significant differences in clinical presentation between enterococcal species were found. Polymicrobial infections presented with wound discharge in 88% (31/35) and lack of fever in 80% (28/35) of episodes, which was significantly more often than monomicrobial infections (25% and 50%, *p*<0.001 and *p*=0.03 respectively). In six cases there was monomicrobial growth from a single sample. Early infections accounted for 34 (62%), delayed for 11 (20%) and haematogenous for 10 (18%) episodes. Of the 10 cases with acute hematogenous infections, two had colon tumors, and one patient each had infective endocarditis, urinary infection and cholecystitis. In 5/10 hematogenous cases no primary focus was identified. 48 episodes were new PJI-episodes, whereas 7 episodes were ongoing PJIs with other pathogens where enterococci were isolated during the treatment process. At diagnosis the median C-reactive protein levels were 88 mg/L (IQR: 29-126), and the median leukocyte count was 10.1x10^9^ (IQR: 9.2-12.5). Hip joints were infected in 35 (64%), and knee joints in 20 (36%) episodes.

### Treatment and outcome

Treatment analysis was performed on all 55 cases. Surgical treatment was performed in 48 and no surgery in seven episodes. In 40 (73%) episodes debridement with implant retention was initially performed. In four of these, multiple debridements were performed. Implant exchange or removal was needed at a later stage in four of the cases treated with implant retention. 32 cases were subjected to debridement once, as the only surgical treatment. Six cases were treated with implant exchange, of which five were two-stage exchanges and one was a one-stage procedure. In 3/5 two-stage exchanges antibiotic spacers were used. In 12 episodes the initial treatment intention was not to cure the patient, but to control the infection by means of prosthesis removal, amputation or antimicrobial suppression therapy. In 43 episodes the treatment intention was to cure the infection.

Antibiotic treatment with enterococcal activity was administered for a total median time of 96 (IQR: 48-140) days, with intravenous therapy for a median of 14 (IQR: 8-21) days. A variety of antibiotics were used, in many cases several changes were made during the treatment period. Intravenous treatment (i.v.) with an enterococcal-active beta-lactam (ampicillin, imipenem/cilastatin (*E. faecalis* only) or piperacillin/tazobactam) was given in 30 (55%) episodes and i.v. glycopeptides (vancomycin or teikoplanin) in 39 (71%) episodes. Aminoglycosides were only given in 3 episodes, of which 2 were intraarticular infusions. Oral follow-up with beta-lactams was given in 35 (64%) episodes and linezolid in 14 episodes. In 10 episodes rifampicin combinations were used for ≥ 2 weeks (range 19-200 days, median 90 days), of which 8 were poly-microbial infections involving staphylococci (**Table 2**).

The outcome analyses included 49 episodes with failure or event-free follow-up of ≥ one year. Six episodes were excluded of which four were lost to follow-up and two because of death from other causes within one year.

**Figure 2:**
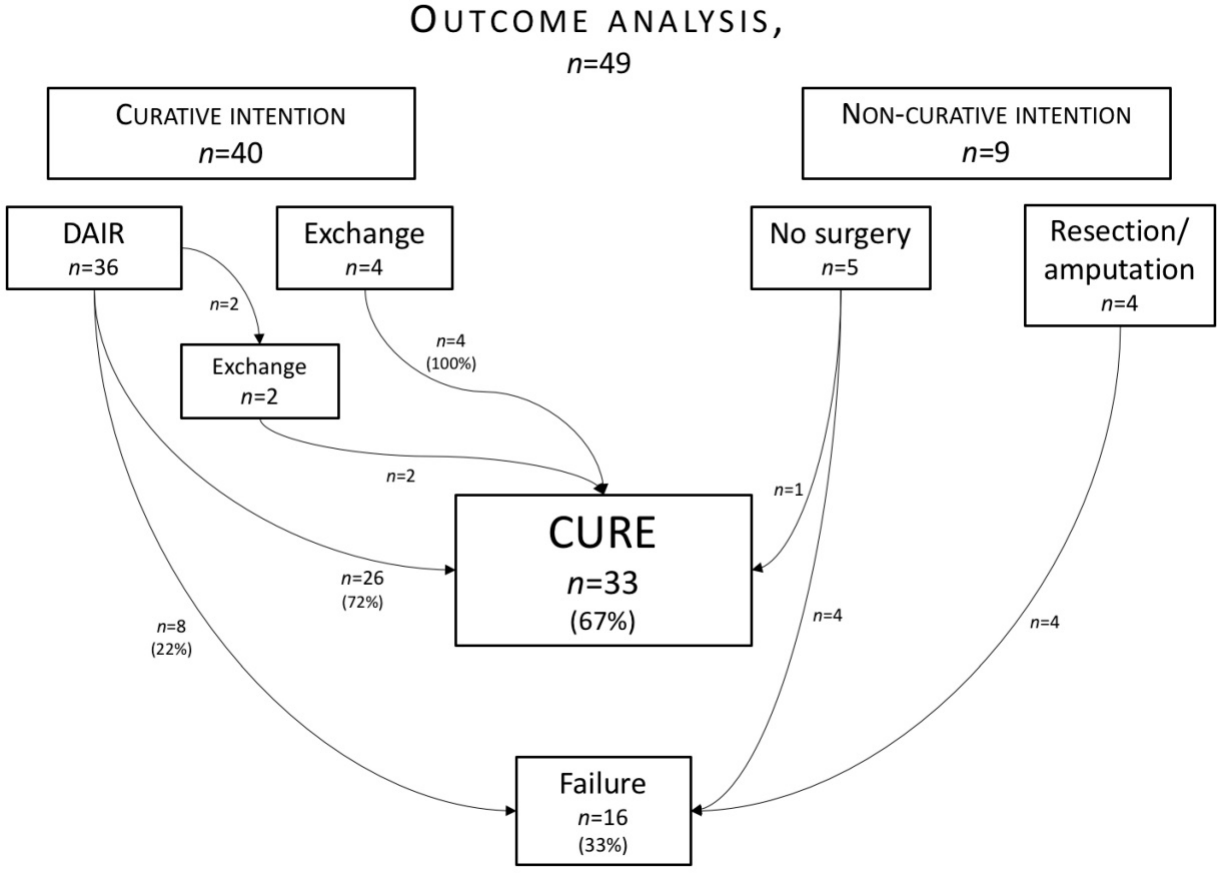
** Outcome analysis and surgical treatment.** In the top row cases are divided into groups according to treatment intention. In the second row the surgical treatment alternatives are reported. Outcome according to treatment is visualized by arrows with corresponding number of cases.

Overall, failure occurred in 16/49 (33%) episodes (chronic suppressive therapy (n=5), resection arthroplasty or amputation (n=6), relapse (n=3), and death due to infection (n=2)), giving an overall cure-rate of 67%. In 40 episodes the treatment had a curative intention, defined as aiming for complete eradication of the infection with preservation of joint function, and among these, 32 (80%) episodes were successfully cured. All 6 episodes treated with implant exchange were successfully cured, while 26/32 (81%) of the episodes which had revision as the last surgical procedure were cured. This difference was not statistically significant **(Table 3)**. When evaluating DAIR as initial surgical procedure, consecutive implant exchange was deemed as failure. Initial DAIR therefore had a cure-rate of 72% (26/36). Overall cure-rate for DAIR-treated cases (including those two that had their implants exchanged) was 28/36 (78%). Details of the surgical procedures and outcome are outlined in **figure 2**.

**Figure 3:**
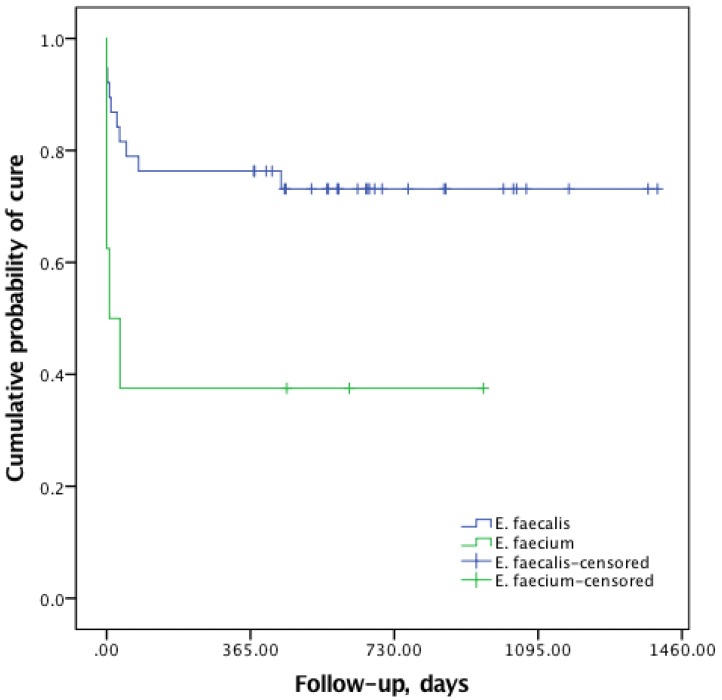
Kaplan-Meier curve of cumulative probability of cure according to enterococcal species (Log-rank test *p*=0.02).

There was no difference in outcome between monomicrobial and polymicrobial infections. When comparing the cumulative probability of cure between infections caused by *E. fecalis* and *E. faecium* we found a significantly worse outcome for the latter (Log-rank test, *p=*0.02, **Fig. 3**). A less favorable outcome was seen in episodes where patients had two or more co-morbidities (*p* < 0.001), and there was also a significant association between higher age and failure (*p* = 0.001). Episodes that were cured presented more often with discharge from the wound (27/33) than episodes that failed (7/16) (*p* = 0.007). The 8 cases treated with rifampicin combination therapy were all cured, which is a significantly higher proportion (*p*=0.04) than those treated with other antibiotic regimens (**Table 3**).

## Discussion

Enterococci are the third most common Gram-positive pathogens isolated in PJI and have been associated with poor outcome 7,14. In recent years there has been a rising interest in enterococcal PJI, but optimal treatment of these infections still remains elusive.

The demographic characteristics of the patient population in our study are well in line with those in other studies, with advanced age and a high frequency of other morbidities.

In accordance with previous reports 7,14,15, we found a high proportion of polymicrobial infections (35/55, 64%). In these previous studies polymicrobial infections have been associated with worse outcomes, but this is not supported by our results. As in the study by Tornero et al. 7, infections caused by *E. faecium* had a worse prognosis. These failures occurred early in the treatment process due to non-curative interventions (resection arthroplasty, amputation or chronic suppression). Bacterial properties, lack of antimicrobial treatment options as well as the disease burden in the affected population may explain this finding.

Over-all cure in our study was 67%, indicating similar or better results than in previous reports. However, definitions for cure vary between studies, impeding comparisons. In our study many of the failed cases were given up early in the treatment process, often due to advanced age or complicating co-morbidities, and these patients were directed to resection arthroplasty, amputation or chronic suppression with antibiotics (Fig 2). This finding is in accordance with results from the study of El Helou et al. 13, but in the study by Tornero et al. 7 these patients were not separately reported. We found that all cases treated with implant exchanges (6/6, 100%) were cured, of which 2 had previous debridements. This is well above the cure rate reported by Tornero 7 and Kheir 14, but in line with the finding of El Helou 13, where 94% of patients treated with 2-stage-exchanges were cured. The El Helou study included only monomicrobial enterococcal PJIs, whereas the infections in our study treated with exchange of prosthesis were predominantly polymicrobial.

An interesting finding in this study was that a majority of the cases (39/55, 71%) were treated with debridement as initial surgery. This reflects the panorama of infection, where early onset and hematogenous infections comprised 80% of all cases. All cases except one with early-onset PJI had debridement as initial surgery, and of these 83% were cured. Adherence to the time-limits outlined in international guidelines for debridement in this group were good (median 21 days, IQR 17-28), possibly explaining some of the results 5,11. These results support the suggestions in the reports by Duijf 15 and Tornero 16, that standardized treatment algorithms are essential, and that adequate results can be achieved with strict adherence. We found no support for the suggestions that initial debridement could jeopardize the treatment results if implant exchange is needed later on. However only few cases had this course of treatment. When comparing with other common pathogens, we found that the cure rate of 72% for DAIR-treated cases was well in line with treatment outcomes for staphylococci (55-88%) and streptococci (75-84%) 9,10,17,18. However, we did not compare PJIs caused by these pathogens and we found no other studies comparing enterococcal PJI with staphylococcal or streptococcal PJI either.

Antimicrobial treatments of enterococcal infections have largely relied on the use of broad-spectrum beta-lactams, such as ampicillin, or glycopeptides. In our study many cases received both glycopeptides and beta-lactams in the initial phase of the treatment, precluding comparative analysis. Traditionally an aminoglycoside has often been added, but recent studies have failed to demonstrate any benefits from this 7,13. The use of aminoglycosides in our cohort was, however, uncommon, and therefore not evaluable. The ampicillin-ceftriaxon-combination is now a recommended treatment option in the setting of enterococcal endocarditis, with interesting study results concerning enterococcal PJI 19,20. This combination was not common in our cohort and was not evaluated in our study.

In accordance with the large multi-center study by Tornero we found a tendency toward better outcome with the use of rifampicin-combination therapy 7. The well-known biofilm acting properties of rifampicin provide a theoretical base for this result, and *in vitro* studies may support the effect of rifampicin-combinations on enterococcal biofilms 21,22. However, all cases except one in our study were given rifampicin combination therapy directed towards co-infections with staphylococci together with amoxicillin, a subgroup with a lower frequency of hematogenous infections. Thus no conclusion can be drawn on possible rifampicin effect specifically against enterococci. There may be a place for rifampicin treatment of enterococcal PJI in the future, but more studies are needed.

The present study is, to our knowledge, the largest population-based consecutive case series of enterococcal PJI. The population-based study-design decreases the risk for selection bias present in studies performed at tertiary centers and constitutes the main strength of the study. The principal limitation is the retrospective nature of the work. Many physicians were involved in treating the patients, and although guideline adherence seems to have been fairly appropriate, we cannot rule out the possibility of personal preference in decision-making. This includes decisions on chronic suppression, with the possibility of case ascertainment bias in the failure group. The final number of cases analyzed, particularly in the outcome analysis, is small, and constitutes a limitation. The follow-up time of one year could be too short to capture all relapses, which is another limitation. Furthermore, in analyzing the data, there was lack of power to perform multivariable analyses, and analyses are presented without corrections for mass-significance.

## Conclusions

We have shown that enterococci should not necessarily be regarded as difficult-to-treat since, when curative treatment is possible; cure was achieved in 80% of the cases. DAIR treatment can be an adequate alternative with a cure-rate of more than 70%.

## Author contributions

OT, PÅ and MR designed the study. Data was collected by OT and analyzed by all authors. OT prepared the manuscript, which was edited by all the authors.

## Figures and Tables

**Table 1:** Clinical and microbiological features of patients with enterococcal PJI.

	All episodes n=55
	n (%) Median (IQR)
Number of episodes	55 (100)
Age	77 (74-87)
Female sex	24 (44)
Co-morbidities	
Ischemic heart disease	14 (26)
Diabetes mellitus	14 (26)
Malignancy (past or present)	10 (18)
Chronic renal insufficiency	9 (16)
COPD^1^	0 (0)
Cirrhosis of the liver	0 (0)
≥ 1 co-morbidity	28 (51)
≥ 2 co-morbidities	13 (24)
Species	
*E. faecalis*	45 (82)
*E. faecium*	8 (15)
*E. caselliflavus*	1 (2)
*E. faecalis + E. faecium*	1 (2)
Polymicrobial infections	35 (64%)
Co-infecting pathogens	
*Staphylococcus aureus*	14
CoNS	17
Gram negative rods	8
Other†	7
Site of implant	
Hip	35 (64)
Knee	20 (36)
Type of infection	
Early	34 (62)
Delayed	11 (20)
Heamatogenous	10 (18)
Episode-type	
New episode	48 (87)
Ongoing episode	7 (13)
Positive blood culture	6 (11)
Fever ≥ 38ºC	17 (31)
Wound discharge	36 (65)
CRP^3^ (mg/L)	88 (29-126)
WBC^4^ x 10^9^/L	10,1 (9,2-12,5)
WBC^4^ > 8.8 x 10^9^/L^5^	31 (56)

†*B. cereus*, n=2, *B. fragilis*, n=1, *Cl. perfringens*, n=2, Group G streptococcus, n=1, *Neisseria sp*, n=1. ^1^COPD= Chronic obstructive pulmonary disease. ^2^CoNS= Coagulase negative staphylococci. ^3^CRP= C-reactive protein. ^4^WBC= White blood cell count. Missing data on 10 cases. ^5^8.8 x 10^9^ cells/L = upper limit of reference interval.

**Table 2:** Details of diagnosis and treatment of enterococcal PJI.

	All episodes, n=55	
	Median (IQR)	n (%)
Age of prosthesis at diagnosis, d^a^	28.5 (19-653)	
Age of prosthesis at first surgical procedure, d^b^	27 (19-169)	
Curative intention of treatment		
Yes		43 (78)
Initial surgical procedure		
No surgery		7 (13)
Debridement		40 (73)
2-stage exchange		4 (7)
Resection arthroplasty or amputation		4 (7)
Final surgical procedure		
No surgery		7 (13)
Debridement		36 (66)
≥ 2 debridements		4 (7)
1-stage exchange		1 (2)
2-stage exchange		5 (9)
Resection Arthroplasty or amputation		6 (11)
		
Intravenous antibiotic treatment, d	14 (8-21)	
Oral antibiotic treatment, d	84 (47-131)	
Total antibiotic treatment, d	95 (48-140)	
Intravenous antibiotic		
Beta-lactam		28 (51)
Glycopeptide		39 (71)
Oral antibiotic		
Beta-lactam		35 (64)
Linezolid		14 (26)
Rifampicin combination^c^		10 (18)

NOTE: d; days. ^a^ Missing data, n=1. ^b^ Variable evaluable in 47 episodes. ^c^ Ciprofloxacin, n=7, clindamycin, n=1, fucidic acid, n=1, linezolid, n=1.

**Table 3:** Univariable analyses of clinical factors according to outcome

	Failure, n=16		Cure, n=33		
Variable	n (%)	Mean (SD)	n (%)	Mean (SD)	p values
Species					
*E. faecalis*	10 (63)		29 (88)		.09
*E. faecium*	5 (31)		3 (9)		
*E. casseliflavus*	0 (0)		1 (3)		
*E. faecalis + faecium*	1 (6)		0 (0)		
Co-morbidity ≥1	9 (56)		15 (45)		.6
Co-morbidity ≥ 2	9 (56)		3 (9)		**.001**
Co-morbidity ≥3	5 (31)		0 (0)		**.002**
Age at diagnosis		83.4 (7.4)		76.3 (6.7)	**.001**
Polymicrobial infection	9 (56)		25 (76)		.2
CRP (mg/L)		127 (122.9)		78 (72.0)	.1
WBC		9.1 (3.4)		11.2 (3.1)	.06
Fever >38º	7 (44)		8 (24)		.2
Discharge	7 (44)		27 (82)		**.01**
					
Type of infection					
Early	5 (31)		26 (79)		.09^a^
Delayed	5 (31)		6 (18)		.2^b^
Heamatogenous	6 (38)		1 (3)		**.001**^c^
Episode type					
New episode	12 (75)		30 (91)		.2
Ongoing episode	4 (25)		3 (9)		
Site of infection					
Hip	12 (75)		18 (55)		.2
Knee	4 (25)		15 (45)		
Initial surgical strategy					
Debridement	8 (50)		28 (85)		.6^†^
2-stage exchange	0 (0)		4 (12)		
RA or amputation	4 (25)		0 (0)		
No surgery	4 (25)		1 (3)		
Final surgical management					
Debridement	6 (38)		26 (79)		.6^†^
Exchange	0 (0)		6 (18)		
RA or amputation	6 (38)		0 (0)		
No surgery	4 (25)		1 (3)		
Antibiotic treatment duration^d^					
≤ 90 days	5 (50)		16 (50)		1.0
> 90 days	5 (50)		16 (50)		
Oral antibiotic^e^					
Beta-lactam	9 (90)		22 (67)		.5
Linezolid	1 (10)		10 (30)		.08
Rifampicin-combination^f^	0 (0)		8 (24)		**.041**
Age of prosthesis at diagnosis					
≤ 2 years	7 (44)		30 (91)		**.001**
> 2 years	9 (56)		3 (9)		

NOTE: CRP; C-reactive Protein, WBC; White Blood Cell Count, RA; Resection Arthroplasty. ^†^ Comparing only Debridement with Exchange ^a^Comparing Early and Delayed ^b^Comparing Delayed and Hematogenous ^c^Comparing Early and Hematogenous ^d^ Variable evaluated in 42 patients. ^e^ Variable evaluated in 43 patients. ^f^ All patients also received beta-lactam.
